# Combining WGCNA and machine learning to construct immune-related EMT patterns to predict HCC prognosis and immune microenvironment

**DOI:** 10.18632/aging.204898

**Published:** 2023-07-21

**Authors:** Yating Sun, Shengfu He, Mingyang Tang, Ding Zhang, Bao Meng, Jiawen Yu, Yanyan Liu, Jiabin Li

**Affiliations:** 1 Department of Infectious Diseases, The First Affiliated Hospital of Anhui Medical University, Hefei, Anhui, China; 2 Anhui Center for Surveillance of Bacterial Resistance, Hefei, Anhui, China; 3 Institute of Bacterial Resistance, Anhui Medical University, Hefei, Anhui, China; 4 Department of Oncology, Anqing First People’s Hospital of Anhui Medical University/Anqing First People’s Hospital of Anhui Province, Anqing, Anhui, China

**Keywords:** EMT, machine learning, WGCNA, HCC, prognosis

## Abstract

Hepatocellular carcinoma (HCC) is a malignancy with a very high mortality rate. Because of its high heterogeneity, there is an urgent need to find biomarkers that accurately predict prognosis. Epithelial-mesenchymal transition (EMT) is closely associated with frequent recurrence and high mortality of HCC. Therefore, it is necessary to comprehensively analyze the prognostic value and immunological properties of EMT gene in HCC. In our study, we performed bioinformatics analysis of the TCGA and ICGC liver cancer cohorts and identified the module genes of immune-associated EMTs (iEMT) by Weighted Gene Co-Expression Network Analysis (WGCNA). Further we used machine learning (support vector machines-recursive feature elimination and Lasso) to identify three central iEMT genes (ARMC9, ADAM15 and STC2) and construct iEMT_score. Subsequently, in the training and validation cohorts, it was demonstrated that the overall survival (OS) of patients in the high iEMT_score group was worse than that of patients in the low iEMT_score group. Based on this, we have constructed a nomogram that is easy for clinicians to use. In addition, our study explored differences in pathway enrichment, immunological properties, and sensitivity to common chemotherapy and targeted drugs in different subgroups of iEMT_score. Finally, we showed through *in vitro* experiments that knockdown of ARMC9 could significantly inhibit the proliferation, migration and invasion of HCC cells BEL7402. Taken together, our findings suggest that iEMT_score is an excellent biomarker for predicting prognosis and provide some new insights for personalized treatment of HCC patients.

## INTRODUCTION

Primary liver cancer is one of the most common malignancies in the world, ranking sixth in incidence among malignancies and third in mortality, causing more than 830,000 deaths each year [1]. Hepatocellular carcinoma (HCC) is a type of primary liver cancer that accounts for the vast majority of primary liver cancers [2]. Risk factors for HCC include chronic HBV or HCV infection, alcoholic liver disease, metabolic liver disease, and long-term exposure to aflatoxin and aristolochic acid [3]. In the early stages of HCC, treatment with therapeutic options such as surgical excision, local ablation, or liver transplantation may be available [3]. Unfortunately, most patients with HCC are found to be in advanced stages of the disease and treatment is limited. Even in developed countries, its 5-year survival rate is only 18% [4]. It is well known that HCC is a highly heterogeneous disease, and accurately predicting the prognosis of patients with HCC is still extremely challenging. Therefore, there is still an urgent need to find biomarkers that can accurately and individually assess and improve the survival time of patients with HCC.

EMT is the process by which epithelial cells are converted into interstitial cells, resulting in high metastatic potential for epithelial cells. Current research suggests that EMT plays a vital role in tumorigenesis, metastasis, immune evasion, and treatment resistance [5, 6]. It has been reported that frequent recurrence and mortality of HCC are closely related to metastasis, and EMT is considered to be the key to metastasis [7]. Previous studies have shown that EMT-related genes are strongly associated with HCC. For example, CAPZA1 can inhibit EMT by remodeling the cytoskeleton, thereby reducing the transfer capacity of HCC cells [8]. NCSTN induces EMT by upregulating ZEB1, thereby facilitating the transfer capacity of HCC cells *in vivo* and *in vitro* [9]. Therefore, it is necessary to comprehensively analyze the EMT gene and construct new biomarkers to accurately predict the prognosis of HCC patients and guide clinical personalized treatment.

The tumor microenvironment (TME) is often defined as a complex and rich multicellular environment that contains immune cells, stromal cells, extracellular matrix, and other secretory factors. Current studies have shown that TME plays a key role in tumor progression and modulating the efficacy of cancer treatment, and immunotherapies targeting TME have been widely used [10]. However, there is a lack of means to determine the composition of TME. The ESTIMATE algorithm is a bioinformatics method to infer TME, so as to obtain immune score and matrix score, thus solving the difficult problem of assessing TME status [11].

In this study, we constructed a weighted gene co-expression network between EMT genes and Immunescore using the WGCNA algorithm to obtain the modules most relevant to Immunescore, and then performed differential expression analysis of EMT genes using limma packets, and combined key module genes and differentially expressed genes to obtain EMT immune-related genes (iEMT). Machine learning SVM-REF was used to eliminate the features of IEMT, followed by LASSO regression analysis to narrow down key iEMT again and construct iEMT_score.

So far, this is the first time that WGCNA and machine learning have systematically evaluated 1872 EMT genes and constructed a new iEMT_score. The model has excellent performance for predicting overall survival (OS) of HCC patients and was validated in TCGA and ICGA cohorts. More importantly, we constructed a nomogram based on iEMT_score, which can be used more easily by clinicians to predict the prognosis of HCC patients. Furthermore, we comprehensively analyzed the relationship between iEMT_score and immune microenvironment. Finally, we identified the oncogenic role of the key model gene ARMC9 in HCC. Taken together, our findings suggest that iEMT_score is an excellent biomarker for predicting OS in HCC patients and has the potential to guide personalized therapy. ARMC9 may be a therapeutic target for HCC.

## MATERIALS AND METHODS

### Data collection

mRNA expression data and clinical data of HCC tissues were downloaded from the TCGA database (https://portal.gdc.cancer.gov/repository). In addition, transcriptomic data and clinical data of HCC patients were downloaded from the ICGC database (https://dcc.icgc.org/projects/LIRI-JP). We excluded patients with missing data. Ultimately, the TCGA cohort included data from 50 paracancerous tissues and 374 cancers. The ICGC cohort included data from 232 HCC patients.

### Collection of EMT-related genes

The EMT-related genes involved in this study were obtained from each of the three datasets. 1. 1184 EMT-related genes were obtained from the dbEMT 2.0 database (http://dbemt.bioinfo-minzhao.org/index.html); 2. “HALLMARK_EPITHELIAL_MESENCHYMAL_TRANSITION” gene set was downloaded from the MSigDB database [12] and 200 EMT-related genes were obtained. 3. 815 EMT-related genes were obtained from the EMTome database (http://www.emtome.org/). Based on this, a total of 1872 EMT-related genes were obtained. Details of EMT genes are detailed in Supplementary Table 1.

### Weighted gene co-expression network analysis (WGCNA)

WGCNA is a powerful bioinformatics algorithm that can efficiently integrate highly correlated genes into the same module and perform correlation analysis between modules and phenotypes [13]. First, based on the mRNA expression profile of the TCGA-HCC cohort, we obtained Immunescore using the ESTIMATE algorithm and set a soft threshold with reference to a previous study [14]. Then, WGCNA analysis was performed to screen out the module genes significantly correlated with the Immunescore, and the module with the largest correlation coefficient was selected for further analysis according to the Pearson correlation coefficient.

### Construction and verification of iEMT_score

The Support Vector Machine (SVM) is a machine learning method with powerful classification capabilities [15] that combine with recursive feature cancellation (RFE) to produce better classification performance [16]. In this study, we used SVM-REF to identify genes that play an important role in iEMT, resulting in 34 key genes. Finally, the overall survival (OS) of patients was included, and the machine learning LASSO algorithm was used to screen out the best iEMT affecting the OS of patients, based on which, iEMT_score was constructed. The iEMT_score formula is calculated as follows: iEMT_score = ARMC9 expression * ARMC9 coefficient + ADAM15 expression * ADAM15 coefficient + STC2 expression * STC2 coefficient. HCC patients are divided into high iEMT_score groups and low iEMT_score groups based on iEMT_score median values. The TCGA cohort serves as the training cohort and the ICGC cohort as the validation cohort. The difference in survival between the two groups of patients was compared using the Kaplan-Meier method.

### Construction of nomogram

First, we included clinical data from patients (age, sex, tumor grade, and stage) and used both univariate and multivariate Cox to explore whether iEMT_score is an independent factor influencing OS in HCC patients. Based on this, we combined iEMT_score and clinical data to construct a nomogram that is easy for clinicians to use, and evaluated the accuracy and stability of the nomogram using ROC curves and calibration curves.

### GSEA, GO and KEGG analysis

“c2.cp.kegg.v7.4.symbols.gmt” is downloaded from the MSigDB database [12]. The limma package analyzed the DEGs of the high iEMT_score group and the low iEMT_score group. GSEA, GO and KEGG analyzes were performed using the R packages “org.hs.eg.db”, “clusterProfiler” and “enrichplot”.

### Somatic mutation status analysis

Somatic mutation information in TCGA-HCC samples is downloaded from the Genomic Data Commons Data Portal (https://portal.gdc.cancer.gov/). This data is processed by the R package “maftools” [17]. We finally showed the top 10 genes that are most prone to mutations in the high iEMT**_**score group and the low iEMT**_**score group, respectively.

### Tumor immunological features of iEMT_score

We assessed tumor immunological features from two aspects. 1. The correlation of iEMT**_**score with 48 immune checkpoints and 24 human leukocyte antigen (HLA) family gene expression was explored. 2. We estimated the infiltration status of immune cells in the sample using seven algorithms, including XCELL, TIMER, QUANTISEQ, MCPCOUNTER, EPIC, CIBERSORT−ABS and CIBERSORT. These results are available on the TIMER2.0 database (http://timer.comp-genomics.org/). We used spearman to analyze the correlation of iEMT**_**score with these immune cells.

### Chemotherapy response prediction

The “oncoPredict” package [18] was used to assess the IC50 values of HCC samples for 6 common targeted drugs and chemotherapy drugs. Then, we use the Wilcoxon test to analyze the IC50 difference between the high iEMT_score group and the low iEMT_score group of these 6 common drugs, and use spearman to analyze the correlation between iEMT_score and the IC50 of these common drugs.

### Cell culture and transient transfection

HCC cell lines BEL7402 and HCCLM3 were a gift from Dr. Dai [19]. All cells were cultured in DMEM medium containing 1% penicillin-streptomycin and 10% fetal bovine serum. Lipofectamine 3000 transfection reagent (Thermo Fisher Scientific, China) was used for transient transfection according to the instructions. The sequence of si-ARMC9 is as follows, sense: CCUGGACUCCAGAGUUAAA; antisense: UUUAACUCUGGAGUCCAGG.

### qRT-PCR analysis

RNAiso Plus reagent (Takara Bio, Japan) is used to extract total RNA from BEL7402 cell and HCCLM3 cell. RNA was back transcribed to cDNA using the PrimeScript™ RT Master Mix (Takara Bio, Japan). Quantification was performed by SYBR Green qPCR Master Mix (Vazyme Bio, China) with β-actin as the internal control. Each PCR reaction is performed in triplicate, with the average value used to calculate the expression level. The target gene primer sequences used in this study are described below: β-actin forward: CCCTGGAGAAGAGCTACGAG; β-actin reverse: GGAAGGAAGGCTGGAAGAGT; ARMC9 forward: GCAAGCCTACATCAGCAATGACC; ARMC9 reverse: CTTCTGCCAGTGACGCAAAAGC.

### Cell counting kit-8 (CCK8) assay and Transwell assay

We seeded 2×10^3^ BEL7402 and HCCLM3 cells in 96-well plates and we seeded 4×10^4^ BEL7402 cells and HCCLM3 in the upper chamber (Corning, USA) containing 250ul serum-free medium. The steps of CCK8 assay and Transwell assay are detailed in previous studies in our laboratory [20].

### Statistical analysis

All statistical analysis is performed on R software (Version 4.1.0). The Wilcoxon test is used for pairwise comparisons. The above sections describe more detailed statistical methods. *P* < 0.05 is considered statistically significant.

## RESULTS

### WGCNA identifies EMT immune-associated key module genes

**Figure 1:**
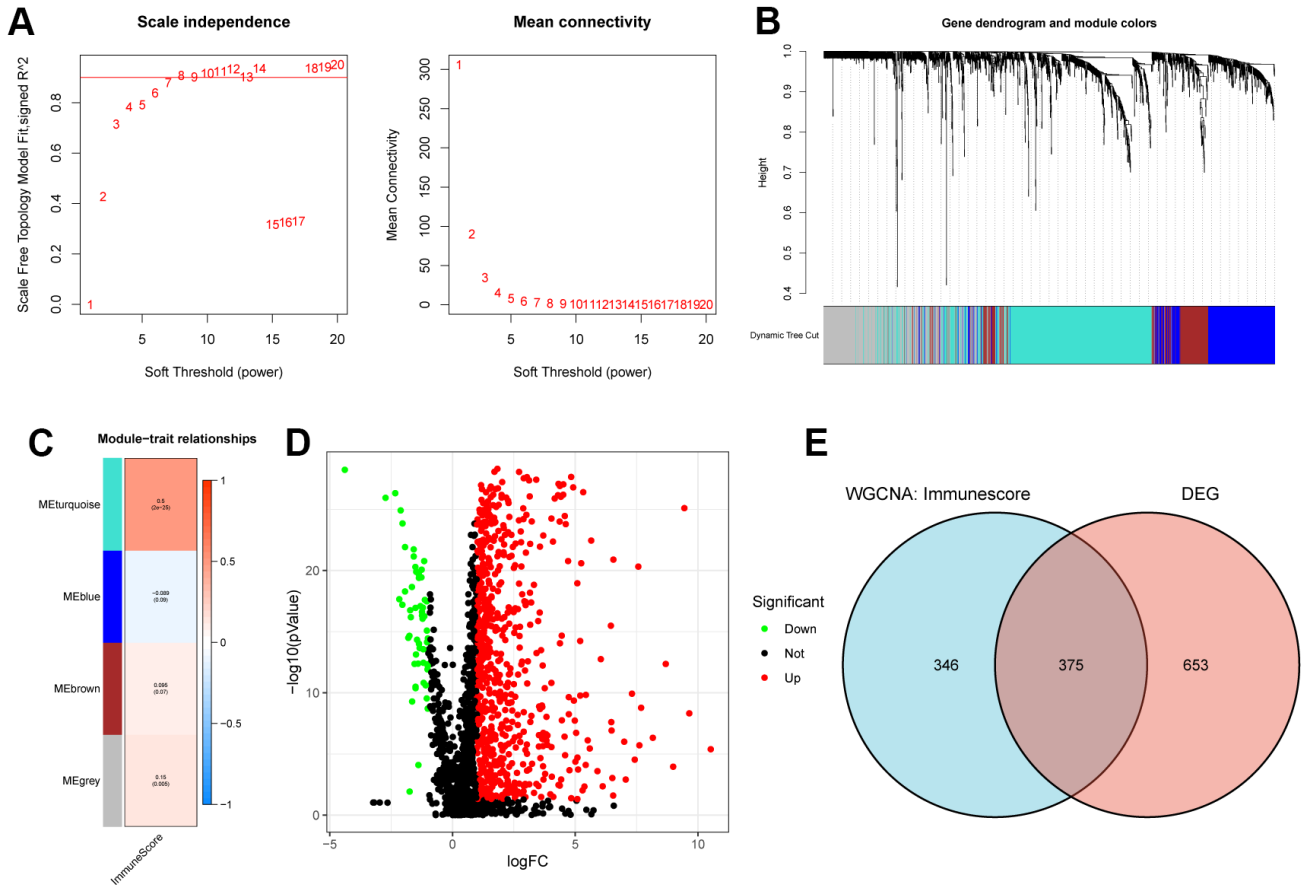
**Acquisition of iEMT gene.** (**A**) Scale independence and average connectivity of the TCGA-HCC cohort. (**B**) Gene dendrogram and modules of the TCGA-HCC cohort. (**C**) Person correlation analysis between co-expressed gene modules and Immunescore in TCGA-HCC cohort. (**D**) Differential analysis volcano map of EMT genes. (**E**) Venn diagram of key module genes and DEGs.

According to the “Materials and Methods” section, we identified 1872 EMT genes. In addition, we used the Immunescore to construct EMT genes-based co-expression network and module for 374 HCC samples using the WGCNA algorithm. By defining module connectivity (Figure 1A), at least 100 genes in the module, Diss Thres is 0.25, and finally WGCNA algorithm determines 4 modules (Figure 1B). According to the thermal spectrum of the correlation between the module and the Immunescore (Figure 1C), the EMT gene correlation coefficient in the MEturquoise module was the highest (721 genes, Cor=0.5, p=2e-25). What’s more, we identified differentially expressed genes (DEG) of the EMT gene in 374 HCC tissues and 50 adjacent normal tissues. As shown in Figure 1D, a total of 1028 genes were differentially expressed (|log_2_foldchange| > 1.5, *P* < 0.05), of which 919 genes were up-regulated and 109 genes expression were down-regulated. To screen for key genes, we screened 375 common genes from the two sets of genes, as shown in the Wayne diagram (Figure 1E). As a result, these 375 genes were identified as immune-associated EMT genes (iEMT) for subsequent analysis.

### Machine learning constructs iEMT_score predicts prognosis in HCC patients

**Figure 2:**
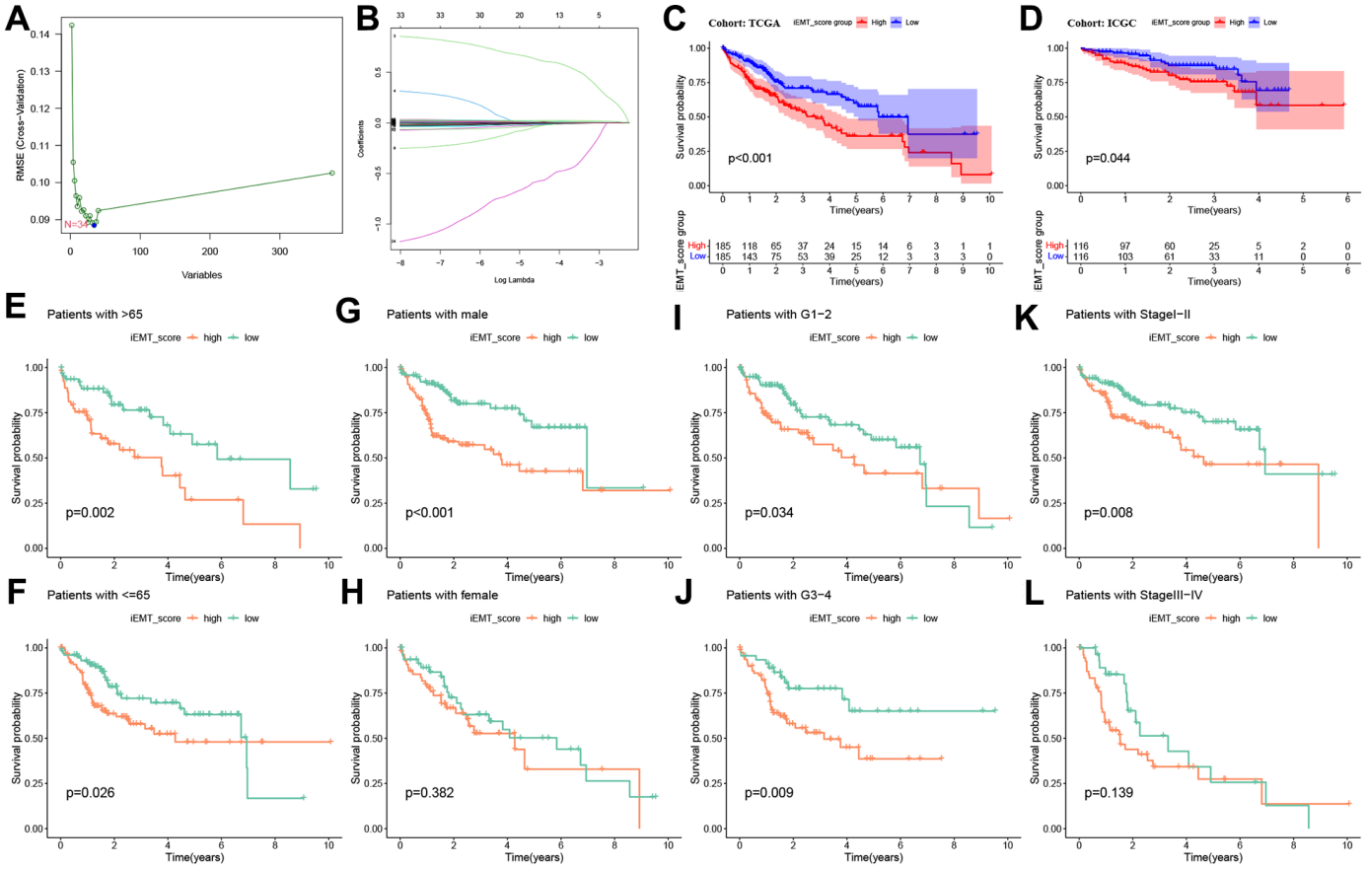
**Machine learning construction iEMT_score and prognostic value verification.** (**A**) SVM-REF algorithm to screen the optimal feature subset. (**B**) Lasso algorithm adjusts feature selection and constructs iEMT_score. (**C**) Kaplan-Meier analysis of iEMT_score subgroups in the TCGA-HCC cohort (training cohort). (**D**) Kaplan-Meier analysis of iEMT_score subgroups in the ICGC-Japan-HCC cohort (validation cohort). Kaplan-Meier survival curve analysis of iEMT_score in different ages (**E**, **F**), genders (**G**, **H**), tumor grades (**I**, **J**) and stages (**K**, **L**).

First, to identify the most important genes in iEMT, we used SVM-REF for further screening. The results showed that the optimal feature subset contains 34 iEMTs (Figure 2A). Then, we included OS data, identified three central genes (ARMC9, ADAM15, STC2, Figure 2B) using Lasso regression analysis, and constructed iEMT_score based on this. We then divided TCGA-HCC patients into high iEMT**_**score group and low iEMT**_**score group based on median iEMT_score. The Kaplan-Meier curve shows that patients in the higher iEMT**_**score group had worse OS than those in the low iEMT**_**score group (Figure 2C). Interestingly, this result was replicated in the validation cohort ICGC-HCC, where patients with low iEMT_score had better OS than those with high IEMT_score (Figure 2D). In addition, we further determined the prognostic value of iEMT_score in HCC patients with different pathological features. The results showed that iEMT_score could not effectively predict the prognosis of HCC patients in women and patients with tumor stages I-II, but showed excellent prognostic ability in the rest of the groups. Collectively, these results suggest that our iEMT_score can effectively predict the prognosis of HCC patients (Figure 2E–2L).

### Construct nomogram based on iEMT_score

**Figure 3:**
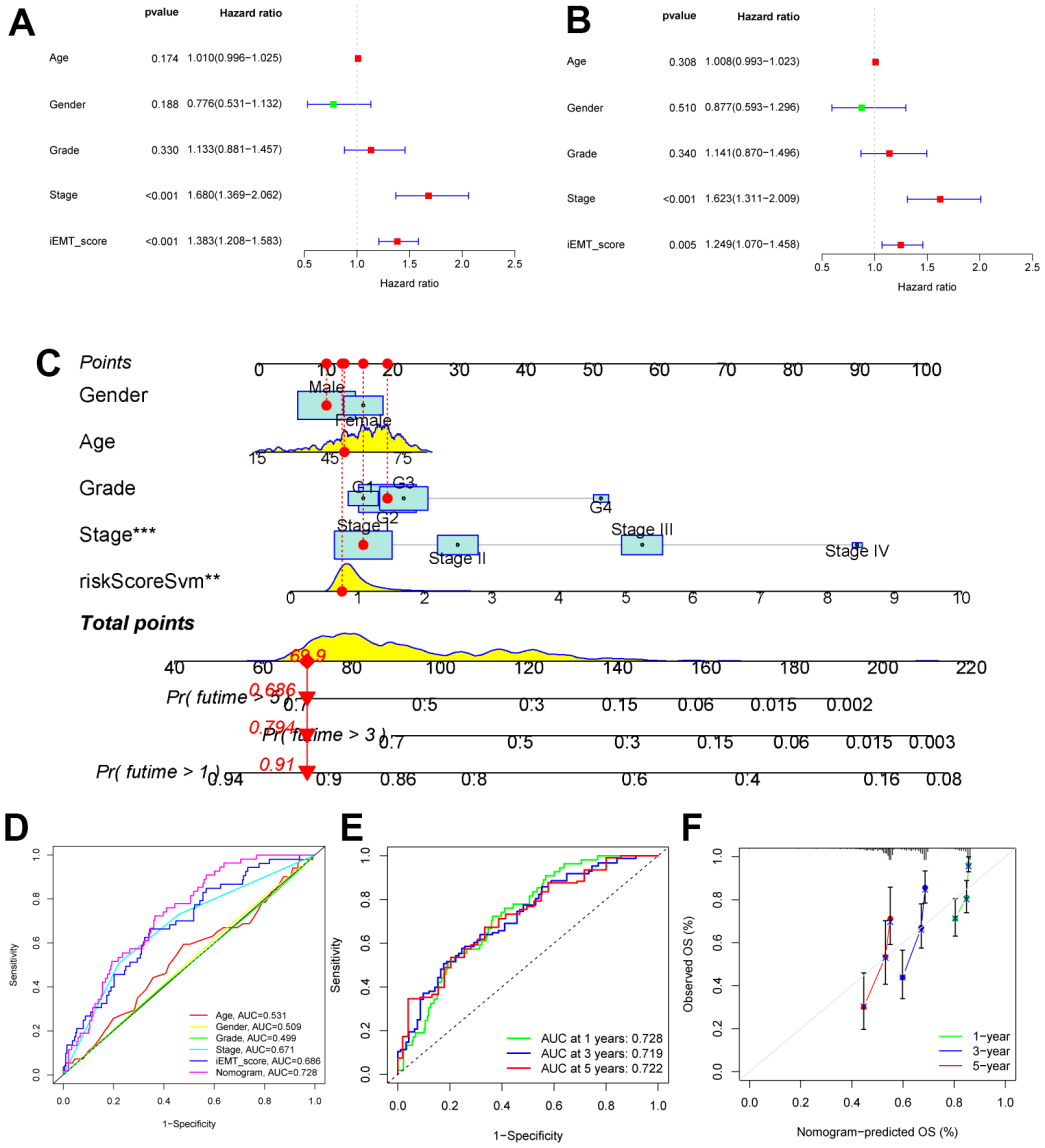
**Construction of nomogram.** (**A**, **B**) Univariate and multivariate Cox analysis of common clinical parameters and iEMT_score. (**C**) Nomogram combining common clinical parameters and iEMT_score. (**D**) ROC analysis of common clinical parameters, iEMT_score and nomogram. (**E**) ROC evaluation of the accuracy of the nomo map in predicting 1-year, 3-year and 5-year OS in HCC patients. (**F**) Calibration curves evaluating the robustness of nomo plots in predicting 1-, 3-, and 5-year OS in HCC patients.

First, we included clinical data from patients in the TCGA-HCC cohort. Univariate cox analysis showed that tumor stage and iEMT**_**score are risk factors affecting OS in HCC patients (Figure 3A). Multivariate Cox regression analysis confirmed that tumor stage and IEMT_score were independent risk factors affecting OS in HCC patients after adjusting for clinicopathological factors (Figure 3B). Subsequently, we constructed a novel nomogram combining clinical parameters and iEMT**_**score (Figure 3C). We first used ROC curve to check the AUC value of each indicator to predict OS in HCC patients, and the results showed that iEMT**_**score was significantly better than other clinical parameters (including tumor stage), and the nomogram constructed based on this further improved the accuracy of predicting OS in HCC patients (Figure 3D). Specifically, the nomogram predicts the AUC values of 1-year, 3-year, and 5-year OS in HCC patients by 0.728, 0.719, and 0.722, respectively, demonstrating good predictive power (Figure 3E). In addition, the calibration curves showed that the nomogram predicted 1-year, 3-year, and 5-year OS values for HCC patients were highly consistent with the actual values (Figure 3F).

### GSEA, GO and KEGG

**Figure 4:**
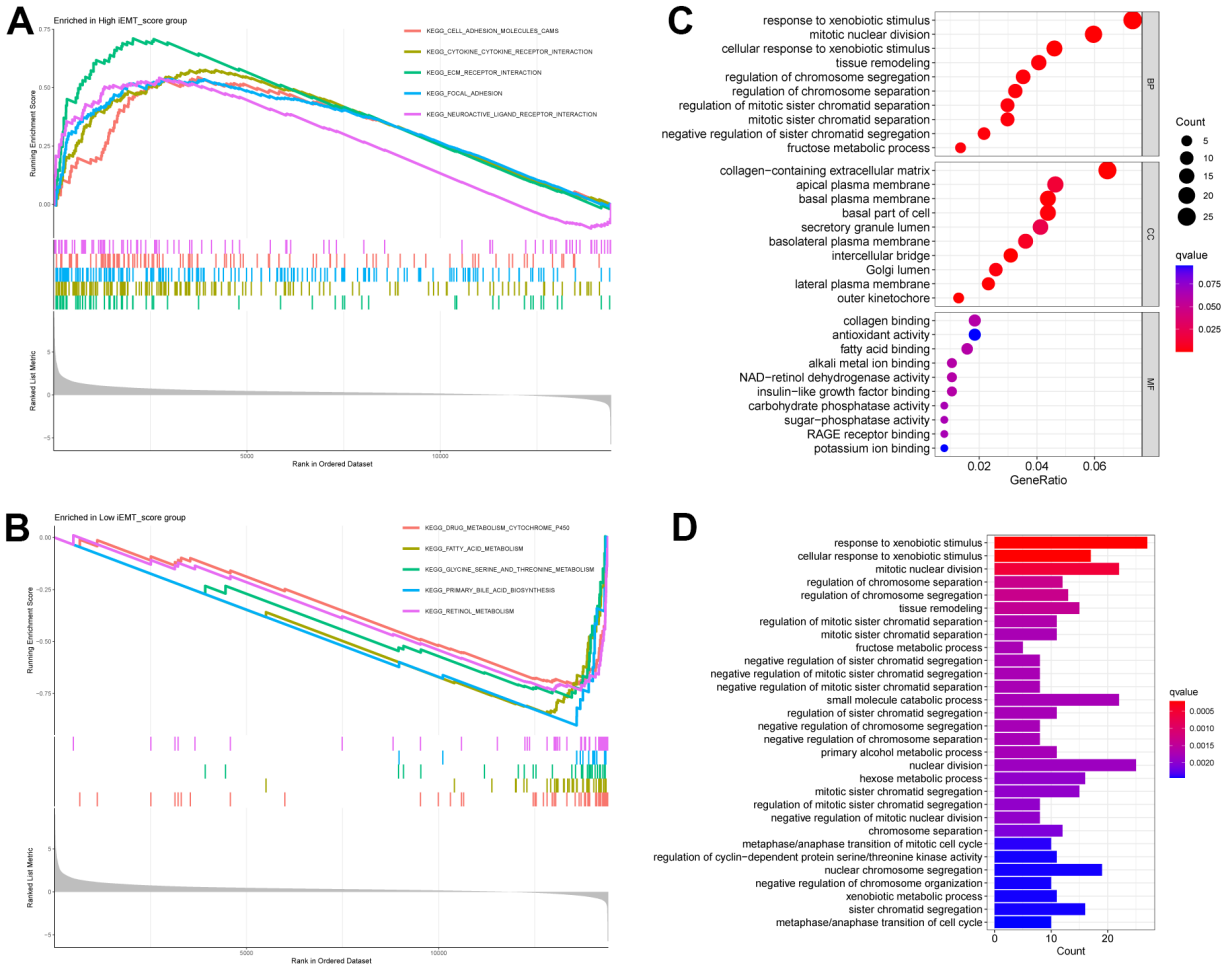
**GSEA of different iEMT_score subgroups.** (**A**) Signaling pathways enriched by High iEMT_score group. (**B**) Signaling pathways enriched by Low iEMT_score group. (**C**) GO analysis for DEGs. (**D**) KEGG analysis for DEGs.

To further explore the mechanism of action that iEMT_score influences HCC, we divided patients into high iEMT_score group and low iEMT_score group by iEMT_score median and further performed GSEA. The results showed that the signaling pathways affected by the high iEMT_score group were mainly enriched in KEGG CELL ADHESION MOLECULES CAMS, KEGG CYTOKINE CYTOKINE RECEPTOR INTERACTION, KEGG ECM RECEPTOR INTERACTION, KEGG FOCAL ADHESION, KEGG LEISHMANIA INFECTION (Figure 4A). Signaling pathways affected by the low iEMT_score group were mainly enriched in KEGG DRUG METABOLISM CYTOCHROME P450, KEGG FATTY ACID METABOLISM, KEGG GLYCINE SERINE AND THREONINE METABOLISM, KEGG PEROXISOME, KEGG RETINOL METABOLISM (Figure 4B). In addition, we also analyzed the DEGs between the high iEMT_score group and the low expression group (|log2foldchange| > 1, *P* < 0.05), and we obtained a total of 428 DEGs. Based on these genes, we further performed GO analysis and KEGG analysis. GO analysis results showed that DEGs were mainly enriched in: response to xenobiotic stimulus, mitotic nuclear division and other BPs; collagen−containing extracellular matrix, apical plasma membrane and other CCs; collagen binding, antioxidant activity and other MFs (Figure 4C). KEGG results showed that DEGs were mainly enriched in the response to xenobiotic stimulus, nuclear division, etc. (Figure 4D).

### iEMT_score is associated with the immunological properties of HCC

**Figure 5:**
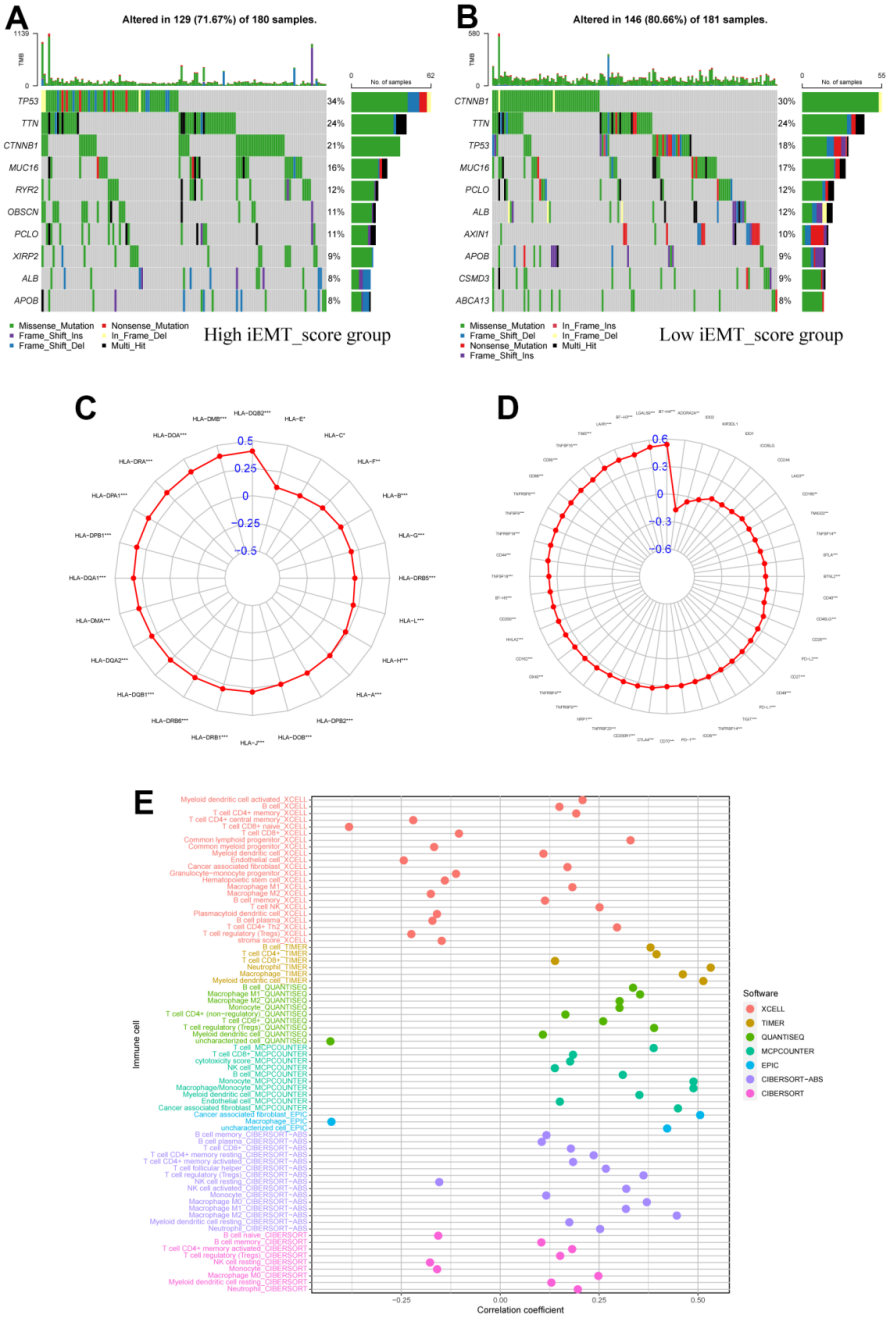
**Immunological properties of different iEMT_score subgroups.** (**A**) Top 10 mutation-prone genes in High iEMT_score group. (**B**) Top 10 mutation-prone genes in Low iEMT_score group. (**C**) Correlation between iEMT_score and expression of 48 immune checkpoint molecules. (**D**) Correlation between iEMT_score and 24 HLA family gene expressions. (**E**) Correlation analysis between iEMT_score and immune cell infiltration.

First, we analyzed somatic mutation and identified the 10 genes with the most common somatic mutation in different iEMT_score subgroups. The mutation rates of TP53, TTN, CTNNB1 and MUC16 were higher than 15% in both groups. TP53 mutation was more common in the high iEMT_score group, where CTNNB1 mutation was more common in the low iEMT_score group (Figure 5A, 5B). Next, we also explored the correlation between iEMT_score and gene expression of 48 immune checkpoints and 24 HLA families. According to Spearson analysis, the expression of 42 immune checkpoint genes and 24 HLA family genes was positively correlated with iEMT_score, and 1 immune checkpoint gene expression (ADORA2A) was negatively correlated with iEMT_score (Figure 5C, 5D). Overall, iEMT_score was positively correlated with gene expression at most immune checkpoints and gene expression in all HLA families. In addition, we investigated the correlation between immune cell infiltration levels and iEMT_score as estimated by XCELL, TIMER, QUANTISEQ, MCPCOUNTER, EPIC, CIBERSORT−ABS and CIBERSORT. After integrating the results, the iEMT_score was positively correlated with immune infiltration of B cells, neutrophils, dendritic cells, cancer-associated fibroblasts, and M2 macrophages (Figure 5E).

### Chemotherapy sensitivity between different iEMT_score subgroups

**Figure 6:**
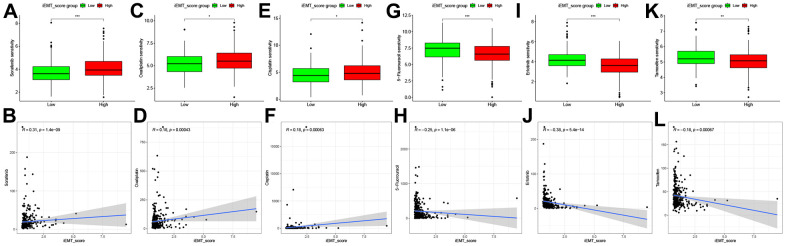
**Correlation analysis between 6 commonly used drugs and iEMT_score.** (**A**, **B**) Correlation analysis between Sorafenib and iEMT_score. (**C**, **D**) Correlation analysis between Oxaliplatin and iEMT_score. (**E**, **F**) Correlation between Cisplatin and iEMT_score analysis. (**G**, **H**) Correlation analysis between 5-Fluorouracil and iEMT_score. (**I**, **J**) Correlation analysis between Erlotinib and iEMT_score. (**K**, **L**) Correlation analysis between Tamoxifen and iEMT_score.

Postoperative targeted therapy and chemotherapy are critical for HCC patients, and Sorafenib remains the first-line therapy for targeted therapy in patients with advanced HCC [21]. Therefore, we explored the correlation between Sorafenib IC50 value and iEMT_score, and the results showed that patients in low iEMT_score group were more sensitive to Sorafenib (Figure 6A, 6B). In addition, we also analyzed the sensitivity of other common targeted drugs and chemotherapy drugs to different iEMT_score subgroups. Patients in the low iEMT_score group were more sensitive to Oxaliplatin and Cisplatin, while patients with high iEMT_score were more sensitive to 5-Fluorouracil, Erlotinib and Tamoxifen (Figure 6C–6L).

### Knockdown of ARMC9 significantly inhibited the proliferation, migration and invasion of the HCC cell line HCC-LM3

**Figure 7:**
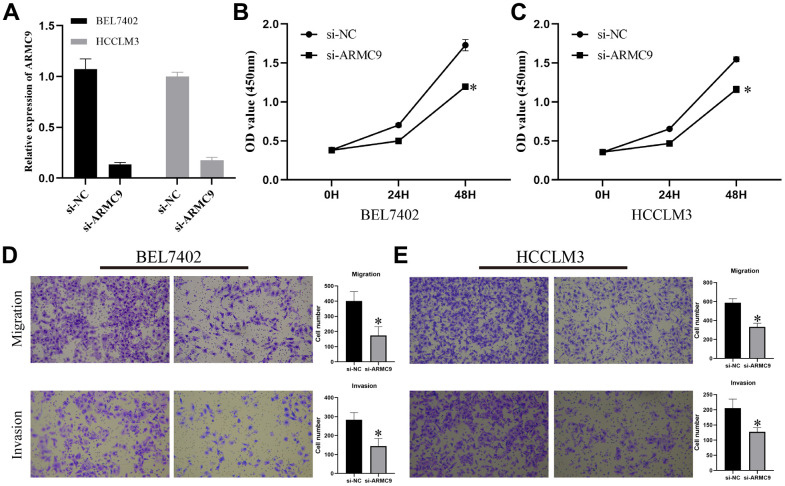
**Exploring the oncogenic role of ARMC9 in HCC.** (**A**) Validation of the knockdown efficiency of si-ARMC9 in BEL7402 and HCCLM3. (**B**, **C**) The effect of ARMC9 knockdown on the proliferation ability of BEL7402 and HCCLM3 cells was evaluated by CCK8 assay. (**D**, **E**) The effect of ARMC9 knockdown on the migration and invasion abilities of BEL7402 and HCCLM3 cells was evaluated by Transwell assay.

To gain insight into the *in vitro* function of CTSA in HCC, we characterized the oncogenic phenotype of BEL-7402 (si-ARMC9) by ARMC9 knockdown. The qPCR results showed that si-CTSA could significantly inhibit the expression of ARMC9 in BEL7402 and HCCLM3 cells (Figure 7A). We investigated the role of ARMC9 in the proliferation of BEL7402 and HCCLM3 cells by the CCK8 method, and the role of ARMC9 in the migration and invasion of BEL7402 and HCCLM3 cells using the Transwell method. CCK8 assay and Transwell assay analysis showed that reducing ARMC9 impaired the proliferation (Figure 7B, 7C), migration and invasion (Figure 7D, 7E) abilities of BEL7402 and HCCLM3 cells.

## DISCUSSION

In this study, for the first time, we comprehensively explored the potential roles of 1872 EMT genes in HCC, and constructed a novel prognostic model (iEMT_score) containing 3 iEMT genes using WGCNA and machine learning. According to the median value of iEMT_score, the patients were divided into high iEMT_score group and low iEMT_score group, and it was found that the OS of patients in the high iEMT_score group was significantly better than that in the low iEMT_score group. These results were validated in the TCGA and ICGC liver cancer cohorts. In addition, our findings showed that iEMT_score was an independent risk factor for predicting OS in HCC patients, and the ROC results showed that iEMT_score was more accurate in predicting OS in HCC patients than common clinical factors (age, gender, tumor grade and stage). These results indicate that iEMT_score has superior performance in predicting OS of HCC patients than common clinical factors, especially tumor stage.

Nomograms are widely used to predict the prognosis of cancer patients and can meet our needs for comprehensive clinical and biological models [22, 23] and widely recognized by clinicians for its friendly digital interface. Although the iEMT_score we generated has a strong role in predicting the OS of HCC patients, there are still great shortcomings in considering biological characteristics alone. In order to make up for this shortcoming, we further constructed a new nomogram combined with clinical indicators. Facts have proved that nomogram has a stronger ability to predict OS of HCC patients than any clinical index or iEMT_score.

It is worth noting that, compared with the prognostic models constructed by other researchers [24–26], we construct iEMT_score with only 3 EMT genes, which is very convenient for clinical application. In the study, ARMC9, ADAM15 and STC2 were identified as the central genes and used to construct IEMT_score. Previous studies have shown that high expression of ADAM15 is associated with poorer OS and promotes proliferation, migration and invasion of HCC cells *in vitro* [27]. STC2 is often highly expressed in HCC patients, which promotes tumor progression through the AKT pathway and is associated with poor overall and disease-specific survival [28]. However, ARMC9 has not been reported in HCC. On the one hand, we verified the expression of three central iEMT genes in HCC tissues at the mRNA level and protein level. Research blanks on ARMC9 in HCC. Therefore, we further explored the biological role of ARMC9 in HCC. The results showed that knockdown of ARMC9 could significantly inhibit the proliferation, migration and invasion of the HCC cell line HCC-LM3. Indicating that ARMC9 may be one of the potential targets for the treatment of HCC patients.

We explored the potential signalling pathways that iEMT_score affects HCC through GSEA analysis. The results showed that the high iEMT_score group was significantly enriched in KEGG CELL ADHESION MOLECULES CAMS, KEGG ECM RECEPTOR INTERACTION, KEGG FOCAL ADHESION, KEGG LEISHMANIA INFECTION and other cancer metastasis-related pathways [29, 30]. This provides partial insights into how iEMT_score affects HCC progression and prognosis. Furthermore, we further understand the immunological properties of iEMT_score subgroups. First, we investigated somatic gene mutations in different iEMT_score subgroups. The largest difference in mutations between groups was that the TP53 gene was more common in the high iEMT_score group than in the low iEMT_score group (34% vs 18%). Previous studies have shown that TP53 is not only the most frequently mutated gene in HCC, but also can affect the progression and prognosis of HCC patients [31]. HCC patients with TP53 mutations have shorter OS and disease-free survival [32]. Therefore, consistent with our survival results, patients in the high iEMT_score group with high TP53 mutations had shorter OS than patients with low TP53 mutations. Second, previous studies have shown that immune checkpoint molecules and HLA family genes are potential predictive biomarkers of response to immunotherapy [33, 34]. Therefore, this study explored the correlation of iEMT_score with 48 immune checkpoint molecules and 24 HLA family genes. The results showed that iEMT_score was correlated with the expression of most immune checkpoint molecules and all HLA family genes. Of course, including (PD-1, PD-L1, CTLA4 and HLA-G) and other classic biomarkers [35–38]. It was closely related to traditional classic biomarkers, implying that iEMT_score is a potential predictive biomarker for ICI response. Finally, we further explore the crosstalk between iEMT_score and TME. We used 7 common algorithms to comprehensively present the immune cell composition of HCC patient tumor tissue. By integrating and analyzing these results, we found that the immune infiltration of iEMT_score B cells, Neutrophil, Myeloid dendritic cell, Cancer associated fibroblast and Macrophage M2 was positively correlated. The role of B cells in HCC remains controversial, with several studies illustrating different results [39, 40]. Elevation of Neutrophil generally correlates with worse OS in most cancers [41], including liver cancer [42]. Likewise, previous studies have shown that tumor progression-promoting CAF and Macrophage M2 are associated with poor prognosis in HCC patients [43, 44]. These partly explain the reason why the prognosis of HCC patients with High iEMT_score is worse.

The frequent emergence of drug resistance seriously impairs the survival time of HCC patients, which greatly troubles clinicians in their treatment options [21, 45]. On the other hand, the new use of old drugs has become an important strategy for the development of anticancer drugs due to its advantages of high drug safety and low cost. Therefore, a comprehensive analysis of drug sensitivity in HCC patients is necessary. In this study, we used “oncoPredict” to estimate the sensitivity of TCGA-HCC cohort patients to 6 common chemotherapy and targeted therapy drugs, including Sorafenib, Oxaliplatin, Cisplatin, 5-Fluorouracil etc. The differences in the sensitivity of different iEMT_score subgroups to 6 chemotherapy and targeted therapy drugs were also explored, which provided some theoretical basis for the personalized treatment of HCC patients.

In conclusion, our study constructed a robust prognostic model that included 3 iEMT genes. The model was shown to accurately predict OS in HCC patients in the training cohort (TCGA) and validation cohort (ICGC). In addition, we also explored the immunological properties of different iEMT_score subgroups, providing some new insights for the individualized treatment of HCC patients. Finally, the oncogenic role of ARMC9 in HCC was demonstrated.

## Supplementary Material

Supplementary Table 1
